# Production of a novel medium chain length poly(3‐hydroxyalkanoate) using unprocessed biodiesel waste and its evaluation as a tissue engineering scaffold

**DOI:** 10.1111/1751-7915.12782

**Published:** 2017-09-14

**Authors:** Pooja Basnett, Barbara Lukasiewicz, Elena Marcello, Harpreet K. Gura, Jonathan C. Knowles, Ipsita Roy

**Affiliations:** ^1^ Faculty of Science and Technology University of Westminster London UK; ^2^ Aarhus University Denmark; ^3^ Eastman Dental Institute University College London London UK; ^4^ Department of Nanobiomedical Science & BK21 Plus NBM Global Research Center for Regenerative Medicine Dankook University Cheonan 330‐714 Republic of Korea

## Abstract

This study demonstrated the utilization of unprocessed biodiesel waste as a carbon feedstock for *Pseudomonas mendocina* CH50, for the production of PHAs. A PHA yield of 39.5% CDM was obtained using 5% (v/v) biodiesel waste substrate. Chemical analysis confirmed that the polymer produced was poly(3‐hydroxyhexanoate‐*co*‐3‐hydroxyoctanoate‐*co‐*3‐hydroxydecanoate‐*co*‐3‐hydroxydodecanoate) or P(3HHx‐3HO‐3HD‐3HDD). P(3HHx‐3HO‐3HD‐3HDD) was further characterized and evaluated for its use as a tissue engineering scaffold (TES). This study demonstrated that P(3HHx‐3HO‐3HD‐3HDD) was biocompatible with the C2C12 (myoblast) cell line. In fact, the % cell proliferation of C2C12 on the P(3HHx‐3HO‐3HD‐3HDD) scaffold was 72% higher than the standard tissue culture plastic confirming that this novel PHA was indeed a promising new material for soft tissue engineering.

## Introduction

Biomaterials have played a significant role in improving health care over the years. They form an interface with the biological systems to treat diseased tissues or organs while restoring their function (Langer and Tirrell, 2004; Ulery *et al*., 2011). The last two decades have witnessed a major paradigm shift from metals to biocompatible and biodegradable polymers for various medical applications such as the development of therapeutic devices and implants. The use of biodegradable polymer implants circumvents biocompatibility issues and complexities of revision surgeries associated with permanent metal implants (Nair and Laurencin, 2007). Most recently, a scaffold‐based tissue engineering approach has demonstrated remarkable promise to create functional biological substitutes to restore tissue function. The underlying concept of this approach is to culture the patient's cells on a biomimetic scaffold made of an ideal biomaterial and deliver the tissue‐engineered construct into the patient, inducing tissue formation. The scaffold should mimic the native cellular milieu and provide a structural framework to encourage the cells to organize into a functional tissue. Natural biopolymers are highly biocompatible, biodegradable and can be produced using renewable resources making them an ideal biomaterial for scaffold fabrication (Yang *et al*., 2001). One such family of natural biodegradable polymers that has gained fresh impetus in recent years is the polyhydroxyalkanotes.

**Figure 1:**
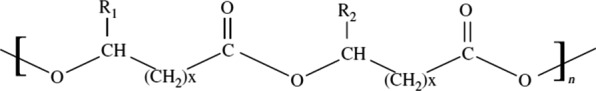
General structure of PHA (Akaraonye *et al*., 2010).

Polyhydroxyalkanotes (PHAs) are biodegradable polymers or bioplastics with a great structural diversity, produced by bacterial fermentation, normally under nutrient limiting conditions. They accumulate within the bacteria as intracellular storage compounds and can be degraded into carbon dioxide and water by a wide range of microorganisms that produce extracellular PHA depolymerases (Khanna and Srivastava, 2005). PHAs are composed of (R)‐hydroxyalkanoates with diverse number of carbon atoms in the monomers (Fig. 1). The monomeric composition of PHAs is regulated by the metabolic pool present in the bacteria and the substrate specificity of the concerned PHA synthase. Depending on the number of carbon atoms present in their monomeric units, PHAs can be classified into short chain length PHAs (scl‐PHAs) containing three to five carbon atoms or medium chain length PHAs (mcl‐PHAs) containing six to 13 carbon atoms in their monomeric units (Basnett and Roy, 2010).

Properties of PHAs vary based on their type, most scl‐PHAs except for poly(4‐hydroxybutyrate) are brittle and have a high melting temperature, whereas mcl‐PHAs are highly elastomeric in nature; have low melting temperature and low glass transition temperature. PHAs can be either semi‐crystalline (scl‐PHAs and most of mcl‐PHAs) or completely amorphous in nature (some of the mcl‐PHAs). Different PHAs exhibit specific material properties, which can be used for particular applications. Amorphous PHAs are easily identified by differential scanning calorimetry (DSC) as they lack a distinct melting point but have a prominent glass transition temperature. Of all the various types of PHAs that exist, amorphous PHAs have not yet been very well explored. However, recently, Metabolix (Cambridge, Massachusetts, United States) have launched a new line of amorphous PHAs as an additive to PVC and PLA for improved material performance and processability, for application in food packaging (Additives for Polymers, 2013; Additives for Polymers, 2014). Polyhydroxyalkanoates have also been widely studied for a range of biomedical applications especially in the field of tissue engineering and medical devices. Scl‐PHAs, such as P(3HB), P(4HB) and their copolymers have been used for hard tissue regeneration, such as bone tissue engineering or drug delivery systems, whereas mcl‐PHAs, such as P(3HO), have been applied for soft tissue regeneration, such as wound healing or cardiac tissue engineering (Lizarraga‐Valderrama *et al*., 2016). The degradation products of PHAs are non‐acidic in nature and therefore do not evoke an inflammatory response. They degrade via surface erosion, therefore, their degradation is controlled, thereby maintaining the integrity of the scaffold under *in vivo* conditions. Hence, PHAs have emerged as promising potential medical materials (Valappil *et al*., 2006; Rai *et al*., 2011a,b; Ali and Jamil, 2016). Furthermore, PHAs have also been identified as one of the safest bioplastics options for the environment. Life cycle assessment (LCA) is a tool that was developed to determine the ecological effect of a product during its life cycle. LCA highlighted that PHAs were one of the safest options amongst the currently available bioplastics (Álvarez‐Chávez *et al*., 2012). Narodoslawsky *et al*. (2015) have demonstrated the ecological potential of PHAs in their critical review, highlighting the use of industrial and ecological waste products as well as clean energy, such as electricity, for the production of polymers.

Due to their attractive properties such as biodegradability and biocompatibility as well as a surge in the demand for green polymers, there has been considerable interest in the commercialization of PHAs in the recent times. One of the major challenges that have deterred their commercial exploitation is their high production cost. Studies have shown that bioplastics constitute only 0.1% of the total polymer market in Europe (Elvers *et al*., 2016). Researchers have used various strategies to lower the cost of production, which include the use of metabolically engineered strains, various fermentation strategies, efficient downstream processing and transgenic plants. Facts state that the cost of the carbon source utilized in the production of PHAs accounts for 31% of the total production cost (Kim and Rhee, 2000; Marangoni *et al*., 2001, 2002). Therefore, one of the most efficient approaches in making PHA production a cost effective process would be to utilize cheap carbon sources. The choice of biodiesel waste as a cheap feedstock for PHA production has gained momentum in the last few years. Biodiesel waste is known to be rich in glycerol content. With the dramatic increase in the biodiesel production globally, crude glycerol is being generated in large quantities. It is estimated that around 1 tonne of crude glycerol is generated for every 10 tonnes of biodiesel. Purification of this crude glycerol, a by‐product of biodiesel production, is, however, an expensive process (Bormann and Roth, 1999; Ashby *et al*., 2004; Ashby *et al*., 2005). Therefore, direct utilization of the untreated biodiesel waste as a feedstock for the production of PHAs could improve the economical viability of both PHAs as well as the biodiesel industry. In this work, a study was carried out where biodiesel waste (crude glycerol phase) was used as the sole carbon source for the production of novel PHAs. This study followed the pattern of informed waste management where waste biodiesel was converted into a value‐added product. In addition, we have also evaluated the use of the novel PHA produced for biomedical application.

## Results and discussion

### Production of PHAs by *Pseudomonas mendocina* CH50 using 50 g l^ −1^ of biodiesel waste as the sole carbon source

**Figure 2:**
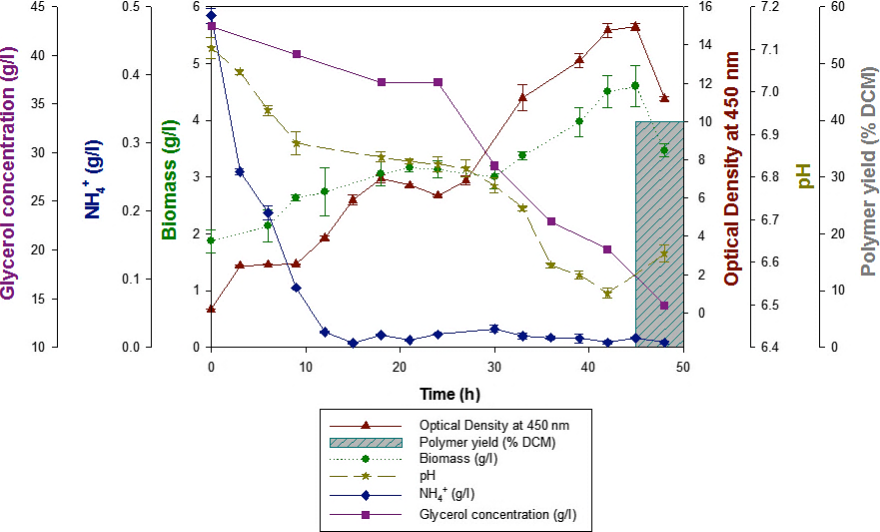
Temporal profile of PHA production by *Pseudomonas mendocina* when cultured using 50 g l^−1^ biodiesel waste as the sole carbon source.

Temporal profiling of the PHA production process in two 5 l bioreactors was carried out as described in the experimental procedures. Various parameters such as the pH, glycerol and nitrogen concentration in the media were monitored (Figure 2).

Optical density (O.D.) increased gradually until 10 h (lag phase) after which it increased rapidly until the end of the fermentation at 48 h, reaching a value of 7.15. The biomass concentration increased during the course of the fermentation and reached its highest value of 4.1 g l^−1^ around 45 h. The starting biomass concentration at 0 h was very high and did not correspond to the optical density value. Samples withdrawn at 0 h were washed with water followed by 10% ethanol. However, media components (impurities) could not be separated from the biomass giving an erroneous value. Characterization of the biodiesel waste to detect its exact composition could provide an insight into the choice of solvent to enable its dissolution. This could help in accurate estimation of the biomass concentration in the initial hours of the fermentation. Apart from 0 h, biomass values were generally low throughout the course of the fermentation. They did not correspond to the optical density values and the glycerol consumption. Previous studies have shown that *P. mendocina* is known to simultaneously produce extracellular alginate during PHA production when cultured using glucose as the carbon source (Guo *et al*., 2011, 2012). More specifically, in a study carried out by Chanasit *et al*., 2016; alginic acid was produced extracellularly when *P. mendocina* PSU was cultured using biodiesel liquid waste as the sole carbon source (Chanasit *et al*., 2016). This could explain the utilization of the substrate; higher optical density and low biomass yield observed in this study. In future, the supernatant could be analysed to detect the production of extracellular polysaccharides.

Nitrogen was the limiting factor in this experiment. It is known that nitrogen limitation coupled with excess carbon triggers PHA production (Khanna and Srivastava, 2005). The concentration of nitrogen reduced from its initial value of 0.5 g l^−1^ to 0.02 g l^−1^ within 12 h of fermentation indicating that a nitrogen‐limiting environment was maintained during the course of the fermentation. However, nitrogen in the form of ammonium ions was measured using phenol hypochlorite method, which only allows quantification of the inorganic nitrogen present in the media. Further increase in the optical density and biomass beyond 12 h indicates that there was an additional source of nitrogen available, probably from the biodiesel waste, which was being utilized by the bacterial cells for their growth and metabolism. Shrivastav *et al*. (2010) reported the production of P(3HB) by novel bacterial strains using jatropha biodiesel waste as a carbon source. Teeka *et al*. (2012) isolated *Novosphingobium* sp. and utilized it for the production of P(3HB) using crude glycerol. They achieved a yield of around 45% cell dry mass (CDM). Chanasit *et al*. (2014) isolated a *Bacillus* sp. ST1C and used it for the production of P(3HB) using biodiesel waste. They achieved a yield of around 72.31% CDM. Several studies indicated that native PHA producers, under nutrient limiting conditions and in the presence of excess glycerol, converted the latter to P(3HB), which is one of the most widely studied PHAs. The pH of the culture medium was adjusted to 7.0 before the inoculation. pH values reduced gradually reaching a value of around 6.6 at the end of the fermentation. pH was not controlled during the fermentation process. This decrease could be due to the production of acid extracellularly into the fermentation media. As described above, several studies have shown that *Pseudomonas* species produce alginic acid simultaneously during PHA production (Guo *et al*., 2011, 2012; Chanasit *et al*., 2016).

**Figure 3:**
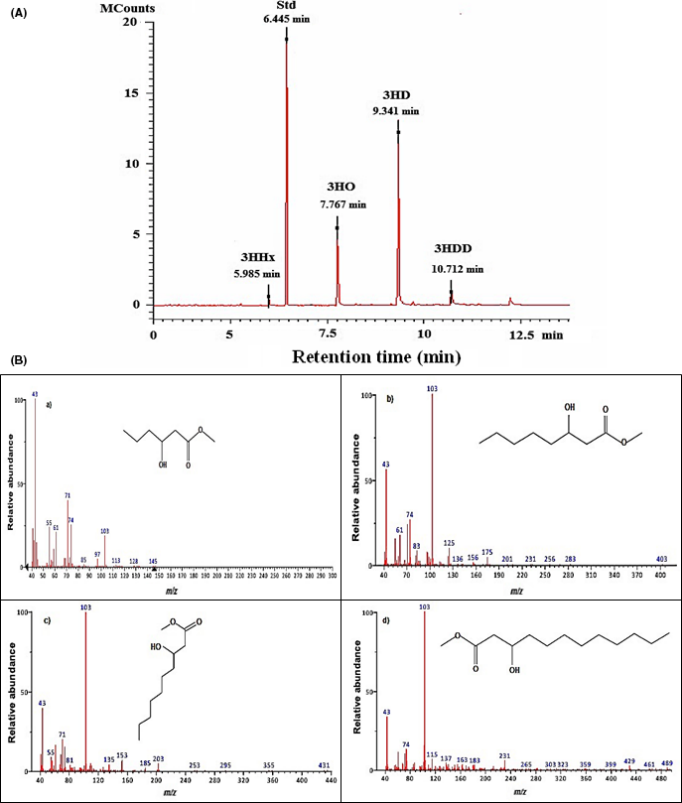
(A) Gas chromatogram and (B) MS spectra of the polymer produced using biodiesel waste as the sole carbon source.

**Figure 4:**
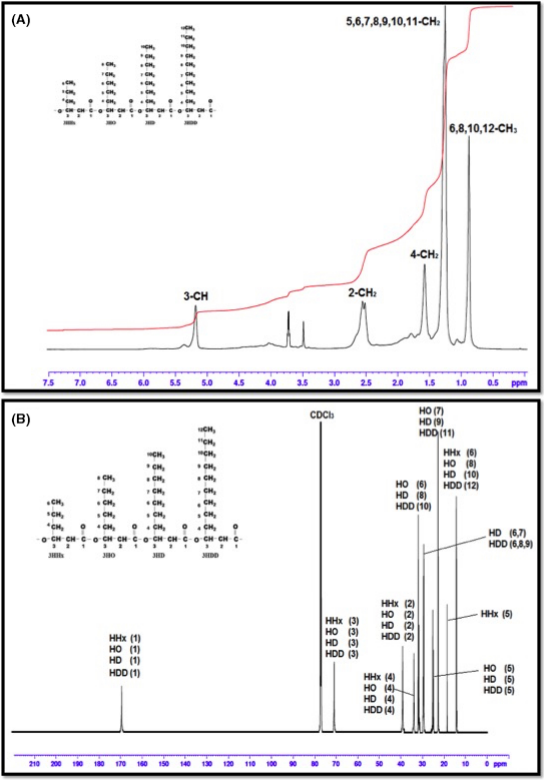
(A) ^1^H NMR spectra and (B): ^13^C spectra of P(3HHx‐co‐3HO‐co‐3HD‐co‐3HDD).

**Figure 5:**
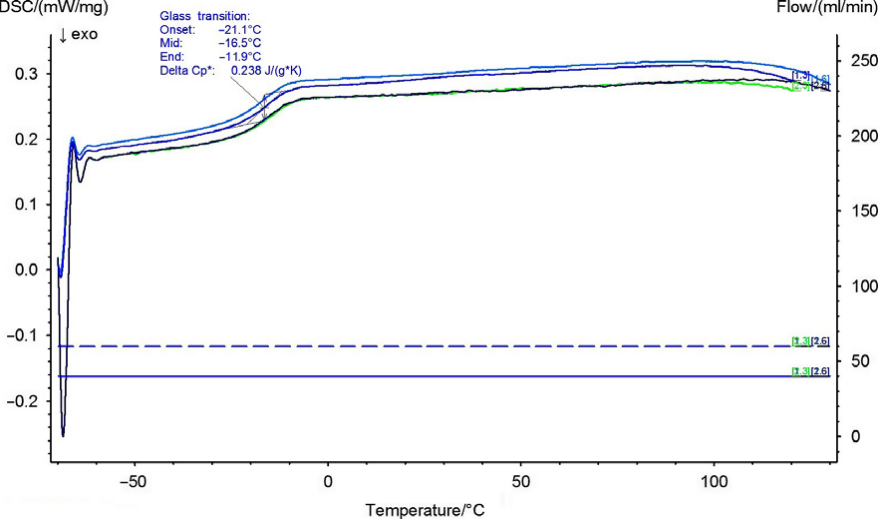
The DSC spectra of P(3HHx‐3HO‐3HD‐3HDD) produced using biodiesel waste as the carbon source.

Glycerol concentration was monitored to investigate the utilization of glycerol by the bacteria. It decreased from its initial concentration of 43 g l^−1^ to 14 g l^−1^ by the end of the fermentation. According to literature, most microorganisms are able to metabolize glycerol for their growth and development. In this particular study, glycerol consumption rate correlated with the increase in the optical density as shown in Fig. 2. The maximum cell dry mass (CDM) obtained was 4 g l^−1^ at 48 h of cultivation. In addition to glycerol, waste by‐products generated from biodiesel production contain several impurities such as methanol, salts, saturated fatty acids such as lauric acid, palmitic acid, stearic acid, relatively high amounts of unsaturated fatty acids such as oleic acid, linoleic acid and alkyl esters (Ribeiro *et al*., 2015). Teeka *et al*. (2012) observed that the novel bacterial strain AIK7 exhibited enhanced growth and higher polymer yield (41% cell dry mass) using biodiesel waste as the sole carbon source compared to pure glycerol. In another study conducted by Ashby *et al*., they found that *Pseudomonas* sp. were able to utilize the free fatty acids and alkyl esters present in biodiesel waste as additional carbon sources to enhance their growth. Ashby *et al*. (2004) observed a yield of 42% CDM when *Pseudomonas corrugata* was grown on biodiesel waste. Poblete‐Castro *et al.,* studied the production of mcl‐PHAs via batch mode by different *Pseudomonas* strains using crude glycerol as the carbon source. *Pseudomonas putida* KT2440 accumulated the highest amount of mcl‐PHAs amongst other *Pseudomonas* strains with a yield of around 34% CDM. This strain was then engineered by deleting the *pha*Z gene responsible for the depolymerization of PHAs, resulting in an improved PHA yield of around 47% CDM (Poblete‐Castro *et al*., 2012). In this study, PHA yield of 39.5% CDM was obtained. This could be further enhanced by employing various optimization strategies such as fed‐batch approaches with different feeding regimes and high cell density cultivation methods (Poblete‐Castro *et al*., 2012). Jiang *et al*. (2013) achieved a yield of about 75.5% CDM using the fed‐batch approach, where three different carbon sources were used as a feed to trigger the synthesis of the polymer with a particular monomer unit composition (Figs 3, 4, 5).

### Characterization of the PHAs produced

#### Gas chromatography mass spectrometry

Monomeric composition of the polymer produced was identified using gas chromatography mass spectrometry (GC‐MS).

The mass spectrum (Figure 3) of the peak at a retention time (*R*_*t*_) of 7.76 min was identical to that of the methyl ester of 3‐hydroxyoctanoic acid, and the peak at a retention time (*R*_*t*_) of 9.34 min was identical to that of the methyl ester of 3‐hydroxydecanoic acid in the MS (NIST) library. The peak at a retention time (*R*_*t*_) of 5.98 min was identical to that of the methyl ester of 3‐hydroxyhexanoic acid, and the peak at a retention time (*R*_*t*_) of 10.7 min was identical to that of the methyl ester of 3‐hydroxydodecanoic acid in the MS (NIST) library, confirming that the polymer produced was a copolymer of 3‐hydroxyhexanoate, 3‐hydroxyoctanoate, 3‐hydroxydecanoate and 3‐hydroxydodecanoate or P(3HHx‐*co*‐3HO‐*co*‐3HD‐*co*‐3HDD) – 2.3 mol% HHx, 27.8 mol% HO, 55.7 mol% HD and 14.2 mol% HDD. Methyl benzoate was used as an internal standard and was represented by the peak at a retention time of 6.4 min. 3‐Hydroxydecanoate (C_10_) was found to be the dominant monomer. Recent studies have shown the monomer content of the PHAs are determined by various enzymes that are capable of metabolizing fatty acids to 3‐hydroxyacyl‐CoA precursors, which are utilized by the PHA synthase enzymes. Several researchers have made similar observations where 3‐hydroxydecanoate was found to be the dominant monomer in the mcl‐PHA copolymer when *Pseudomonas* sp was grown on glycerol, glucose or gluconate (Poblete‐Castro *et al*., 2012, 2013). Studies carried out by Poblete‐Castro *et al*. have shown that *P. putida* LS46 produced mcl‐PHAs with a high amount of 3‐hydroxydecanoate (C_10_) units when cultured in waste glycerol. It has been reported that the *phaG* gene has affinity towards (R)‐3‐hydroxydecanoyl–ACP in both *P. putida* LS46 and *P. putida* KT2440. A high level of *phaG* expression was observed during mcl‐PHA production by *P. putida* LS46 using waste glycerol. It is known that (R)‐3‐hydroxyacyl–ACP is restricted to 10 carbon substrates, and therefore, high amount of 3‐hydroxydecanoate (C_10_) is produced during the biosynthesis of mcl‐PHAs by *Pseudomonas* species (Poblete‐Castro *et al*., 2012, 2013).

Production of P(3HHx‐3HO‐3HD‐3HDD) has been reported earlier. However, the mol% of each monomer obtained in this study is novel. In a study conducted by Cerrone *et al*., mannitol rich ensiled grass press juice (EGPJ) was used a sole carbon source for the production of PHAs under high cell density cultivation. Different strains of *Pseudomonas* sp were investigated for this study. They observed that *P. putida* W619 produced P(3HHx‐3HO‐3HD‐3HDD) – 6 mol% HHx, 19 mol% HO, 68 mol% HD and 5 mol% HDD using EGPJ. In addition, *P. putida* W619 and *Pseudomonas chlororaphis* IMD555 both produced P(3HHx‐3HO‐3HD‐3HDD) using simulated EGPJ. Monomer composition of P(3HHx‐3HO‐3HD‐3HDD) produced by *P. putida* W619 was found to be 1 mol% HHx, 14 mol% HO, 63 mol% HD and 21 mol% HDD, whereas the composition of P(3HHx‐3HO‐3HD‐3HDD) produced by *P. chlororaphis* IMD555 was measured to be 5 mol% HHx, 10 mol% HO, 54 mol% HD and 32 mol% HDD (Cerrone *et al*., 2015). In another study conducted by Mozejko *et al.,* pulsed feeding strategy was employed to enhance the production of mcl‐PHAs from waste rapeseed oil by *Pseudomonas* sp. G101. They identified the monomeric composition of the mcl‐PHA produced as P(3HHx‐3HO‐3HD‐3HDD). The monomeric structure remained unaffected by the cultivation time and feeding strategy, however, the mol% of the monomers varied (Możejko and Ciesielski, 2014). In this particular study, 3‐ hydroxyoctanoate was found to be the dominant monomer. Hence, the mole% of the 3HHx, 3HO, 3HD and 3HDD monomer in P(3HHx‐3HO‐3HD‐3HDD) differed in each organism (summarized in Table 1). None of the previously reported copolymers containing 3HHx, 3HO, 3HD and 3HDD monomers have the same mole % as the mcl‐PHA copolymer produced in this work; hence, a new/novel polymer was produced with a unique monomer content, which would in turn lead to unique material properties.

**Table 1:** Summary of the mol% of P(3HHx‐3HO‐3HD‐3HDD) obtained in various studies

Organism	Carbon source	Mol %			
		3HHx	3HO	3HD	3HDD
*Pseudomonas mendocina* CH50^a^	Biodiesel waste	2.3	27.8	55.7	14.2
*P. putida* W619	EGPJ	6	19	68	5.0
*P. putida* W619	Simulated EGPJ	1	14	63	21.0
*P. chlororaphis* IMD555	Simulated EGPJ	5	10	54	32.0
*Pseudomonas* sp. G101	Waste rapeseed oil‐ (Pulsed feeding at 48 h)	8.24	52.90	35.85	3.01
*Pseudomonas* sp. G101	Waste rapeseed oil(continuous feeding at 48 h)	11.40	48.54	29.82	10.24

a Mol% of P(3HHx‐3HO‐3HD‐3HDD) obtained in this study.

#### Nuclear magnetic resonance

Structural identification of the polymer was confirmed using nuclear magnetic resonance (NMR).

The ^1^H NMR spectrum of P(3HHx‐*co*‐3HO‐*co*‐3HD‐*co*‐3HDD) as shown in Fig. 4A showed signals of the methine protons (CH) at 5.2 ppm demonstrating ‐O‐(CH‐)CH_2_‐ bonding at carbon number 3. The peak present at 0.89 ppm corresponded to the terminal methyl group (CH_3_). The multiplet resonance existing at 2.51 ppm corresponded to the methylene protons (CH_2_), –CH‐(CH_2**)**_‐CO‐ at carbon atom 2. The signal at 1.52 ppm corresponded to the methylene protons ‐CH_2_‐(CH_2_)‐ CH‐ at carbon atom 4. The signal at 1.26 ppm corresponded to all other methylene protons of the saturated side‐chain. CDCl_3_ was used as the solvent, which resulted in a peak at 7.3 ppm. NMR analysis of the mcl‐PHA copolymer produced using biodiesel waste as the sole carbon source also confirmed the GC‐MS data. Hence, the mcl‐PHA was structurally identified to be a P(3HHx‐3HO‐3HD‐3HDD) copolymer.

**Figure 6:**
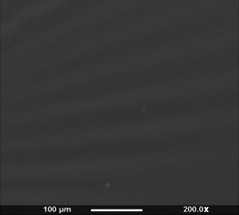
Scanning electron microscopy images of the surface of the solvent cast P(3HHx‐3HO‐3HD‐3HDD) film.

Other biodiesel products were investigated for the production of mcl‐PHAs. The PHAs produced showed different monomeric composition other than the one obtained in this study. Muhr *et al*. (2013a) used saturated biodiesel fractions (SFAE) originating from animal waste lipids for PHA production by *Pseudomonas citronellolis*. P(3HHx‐3HHp‐3HO‐3HN‐3HD) was obtained, and 3‐hydroxyoctanoate and 3‐hydroxydecanoate were the dominant monomers. Another study by the same group investigated the production by *P. chlororaphis* using the same biodiesel product. In this case, P(3HHx‐3HHp‐3HO‐3HN‐3HD‐3HHD) was produced (Muhr *et al*., 2013b).

### Differential scanning calorimetry

Thermal properties of P(3HHx‐3HO‐3HD‐3HDD) including the melting temperature (*T*_m_) and glass transition temperature (*T*_g_) were determined using DSC.

The *T*_g_ (Figure 5) of the copolymer was −21°C, which is typical for the MCL‐PHAs. No melting event was observed during the first and the second heating cycles indicating that the copolymer was amorphous in nature. To confirm this result, further analysis was carried out. The polymer was stored for a period of four weeks before carrying out the DSC studies. There was no melting event confirming that P(3HHx‐3HO‐3HD‐3HDD) was an amorphous polymer. This indicated that the monomer units were randomly oriented at the molecular level. Muangwong *et al*. (2016) carried out an extensive study by employing novel bacteria isolated from the soil such as ASC1, *Acinetobacter* sp. (94.9% similarity); ASC2, *Pseudomonas* sp. (99.2% similarity); ASC3, *Enterobacter* sp. (99.2% similarity); and ASC4, *Bacillus* sp. (98.4% similarity) for the production of mcl‐PHAs using crude glycerol as the carbon source. All the mcl‐PHAs produced were amorphous in nature. Similar findings were reported by Song *et al.,* when *Pseudomonas* sp. strain DR2 was cultivated using waste vegetable oil for mcl‐PHA production. They associated the amorphous nature of the polymer produced with the absence of 3‐hydroxybutyrate monomer units. It is a known fact that the length of the aliphatic chains of a single monomer unit affects the crystallinity of the polymer. Presence of longer aliphatic chains in the monomer units interrupts their arrangement at the molecular level (Song *et al*., 2008). Preusting *et al*. (1990) also concluded that the presence of unsaturated groups interfered with the systematic arrangement of the polymeric chains, resulting in the synthesis of an amorphous polymer.

### Synthesis of P(3HHx‐3HO‐3HD‐3HDD) films by solvent casting and their characterization

P(3HHx‐3HO‐3HD‐3HDD) films were prepared using the solvent cast method. These solvent cast films were thoroughly characterized for their surface properties before being tested for their compatibility with mammalian cells.

### Scanning electron microscopy

The surface topography of the P(3HHx‐3HO‐3HD‐3HDD) film samples was studied using scanning electron microscopy (SEM). Surface topography of the P(3HHx‐3HO‐3HD‐3HDD) film is shown in Fig. 6.

Scanning electron microscopy studies revealed that the surface of the P(3HHx‐3HO‐3HD‐3HDD) film was smooth devoid of pores and protrusions; however, there were undulating areas on the air‐exposed surface of the films, formed during the solvent casting process. This was due to the highly viscous nature of the polymer solution and was observed in all the P(3HHx‐3HO‐3HD‐3HDD) solvent cast films. Previous studies have also shown that mcl‐PHA matrix generally exhibits a smooth appearance (Rai, 2010; Renard *et al*., 2011; Basnett *et al*., 2012). Low crystallization rate of the polymer during the solvent evaporation process would have allowed the polymer to reorganize steadily avoiding the formation of pores and protrusions on the surface (Renard *et al*., 2011).

#### Surface roughness

**Figure 7:**
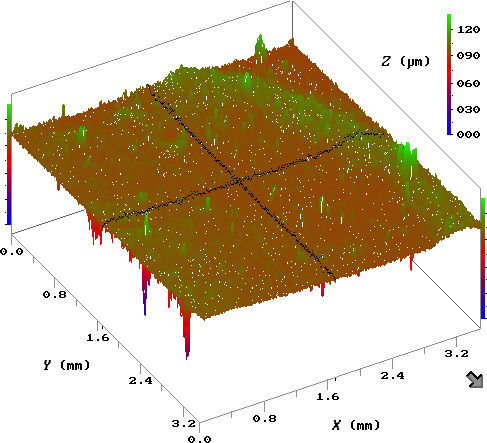
Surface scan of the P(3HHx‐3HO‐3HD‐3HDD) film using the Laser profilometer (*n* = 3).

Surface roughness of the P(3HHx‐3HO‐3HD‐3HDD) film was measured using the laser profilometer. Surface roughness values of the P(3HHx‐3HO‐3HD‐3HDD) film were calculated to be *R*_*q*_ = 1.77 ± 0.29** **μm as shown in Fig. 7.

*R*_*q*_ value of 10 wt% P(3HHx‐3HO‐3HD‐3HDD) film was significantly higher compared to the *R*_*q*_ values obtained for other mcl‐PHA neat films, calculated to be 0.238 μm (Basnett *et al*., 2012) or 0.272 μm (Rai *et al*., 2011a,b). The high *R*_*q*_ value could be due to the uneven surface of the film formed due to the viscous nature of the polymer solution. This was consistent in all the three films that were cast. Surface topography is one of the crucial factors that is considered while evaluating any biomaterial for biomedical applications. Existing paradigm suggests that most cells prefer to attach on a rough surface compared to the smooth surface. Uneven or rough surfaces provide the cells with several adhesion points that allow them to migrate and proliferate (Hallab *et al*., 2001). However, it has been suggested that there is an optimal surface roughness value beyond which it could have a detrimental effect on cell adhesion mechanism (Francis *et al.,* 2016). In addition to surface roughness, two other factors that play an important role in determining the biocompatibility of a material are hydrophilicity and protein adsorption (Hao and Lawrence, 2004).

#### Water contact angle measurements

Static contact angle study was carried out to measure the hydrophobicity of the P(3HHx‐3HO‐3HD‐3HDD) film samples. The tests were carried out in triplicates on three different solvent cast films. Water contact angle (θ) of the P(3HHx‐3HO‐3HD‐3HDD) film was measured to be 77.3 ± 5. It is a known fact that the surface of a material is considered to be hydrophobic if the water contact angle (θ) is higher than 70° (Basnett *et al*., 2012). Therefore, the surface of the P(3HHx‐3HO‐3HD‐3HDD) films was considered to be hydrophobic. Hydrophobicity is associated with the PHAs due to the presence of alkyl pendant groups in their side‐chains. This is in agreement with the other researchers who have observed that the surface of a neat PHA film is inherently hydrophobic. In other studies, the static contact angle values for neat P(3HO) film was within the range of 77.3 ± 1.0 to 101 ± 0.8 (Rai *et al*., 2011b; Bagdadi *et al*., 2016). Contact angle values for P(3HHx‐3HO‐3HD‐3HDD) obtained in this study (77.3 ± 5.0) were within the range described in the literature. However, the θ value (77.3 ± 5) obtained for P(3HHx‐3HO‐3HD‐3HDD) films was lower than that observed for P(3HO) films [θ values (104 ± 2)] as described by Renard *et al*. (2011). This decrease in the θ value could be due to the surface characteristics of the solvent cast P(3HHx‐3HO‐3HD‐3HDD) films (Hao and Lawrence, 2006a,b). In an experiment conducted by Kubiak *et al.,* the influence of surface roughness on the wettability properties of the engineering surfaces was studied. They observed that θ values were strongly affected by the surface roughness. They found that θ values were reduced with intermediate roughness as the water droplet spread along the grooves (Kubiak *et al*., 2011). Hydrophilicity is considered to be a desirable property in a biomaterial or a tissue engineering scaffold. Several studies have demonstrated that most cells prefer to attach and proliferate on hydrophilic surfaces (Hallab *et al*., 2001). Bearing in mind the application of P(3HHx‐3HO‐3HD‐3HDD) films as a tissue engineering scaffold, this decrease in the hydrophobicity of the P(3HHx‐3HO‐3HD‐3HDD) film as compared to the P(3HO) film is a positive outcome. P(3HO) is established as a highly biocompatible PHA (Rai *et al*., 2011a,b). Hence, this result indicates that P(3Hx‐3HO‐3HD‐3HDD) should promote cell adhesion and proliferation as well as P(3HO) and perhaps perform even better than P(3HO).

#### Protein adsorption

Another important factor that influences the biocompatibility of a scaffold is protein adsorption (Rechendorff *et al*., 2006). Bicinchoninic acid assay was carried to quantify the total protein adsorption on the P(3HHx‐3HO‐3HD‐3HDD) film samples. Overall protein adsorbed on the P(3HHx‐3HO‐3HD‐3HDD) film samples was 1.6 ± 0.1 mg cm^−2^. For this particular study, bovine serum albumin (BSA) was the protein of choice. In a detailed study carried out by Jeyachandran *et al*., adsorption of BSA on both hydrophilic and hydrophobic surface was widely investigated. They inferred that the BSA surface coverage was saturated at 53% on the hydrophobic surfaces, whereas on the hydrophilic surfaces, BSA coverage was almost up to 95%. In addition, the interaction of the BSA molecules on the hydrophilic surfaces was much stronger compared to the hydrophobic surfaces (Jeyachandran *et al*., 2009). The amount of protein adsorbed on the P(3HHx‐3HO‐3HD‐3HDD) film samples in this study was significantly higher compared to values reported in the literature for the other PHA films of similar dimension and incubation time of 24 h. For example, BSA adsorption on neat P(3HO) film was previously observed to be 0.083 mg cm^−2^ (Rai, 2010) as compared to the value of 1.6 ± 0.1 mg cm^−2^ observed for the P(3HHx‐3HO‐3HD‐3HDD) film. The difference was significant (**P = 0.0018). This outcome is coherent with the slightly lower θ value followed by the increased *R*_*q*_ value obtained for P(3HHx‐3HO‐3HD‐3HDD) films compared to other neat mcl‐PHA films. This finding thus supports the fact that protein adsorption is higher on hydrophilic and rough surfaces (Hao and Lawrence, 2006a,b). This higher protein adsorption observed on the P(3HHx‐3HO‐3HD‐3HDD) films might confer higher biocompatibility to this novel material.

It is a known fact that mammalian cells are anchorage‐dependent. They attach onto protein‐rich surfaces and proliferate; therefore, protein adsorption is an important phenomenon while considering various biomaterials in TE applications (Chen *et al*., 2009). Sato and Murahara (2004) made an interesting observation that protein adsorption was higher on fabricated surfaces which were rough and less hydrophobic. Several techniques such as laser technology, microcontact printing and photopatterning have been implemented to fabricate the surface of the polymer films to enhance protein adsorption and cell adhesion (Hao and Lawrence, 2006a,b; Chen *et al*., 2009).

#### *In vitro* biocompatibility studies

**Figure 8:**
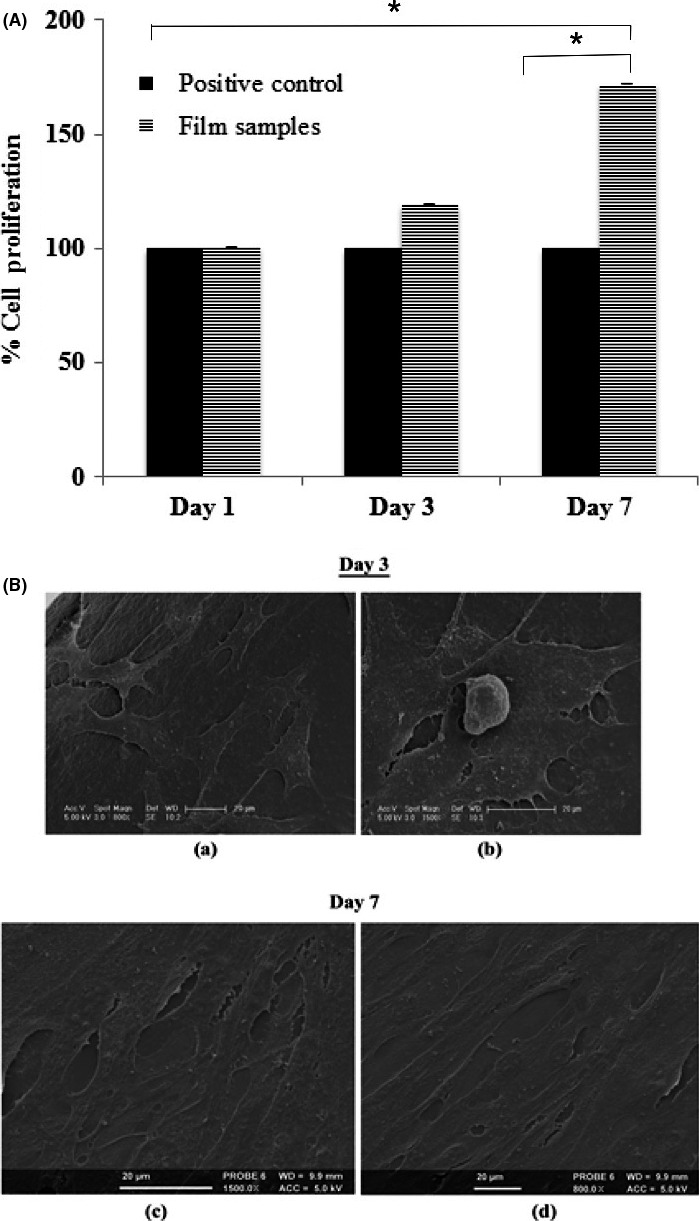
(A) Cell proliferation study of the C2C12 cells on the P(3HHx‐3HO‐3HD‐3HDD) film samples on day 1, 3 and 7 (*n* = 3). All the film samples were measured relative to the C2C12 cells on the tissue culture plate (TCP), which were normalized to 100% (*n* = 3). (B) SEM images of the C2C12 cells on the P(3HHx‐3HO‐3HD‐3HDD) film samples on day 3 (a–b) and 7 (c–d). Size bar: 20 μm.

Preliminary *in vitro* cell culture studies were carried out on P(3HHx‐3HO‐3HD‐3HDD) films using the C2C12 cell line. C2C12, which is a myoblast cell line isolated from mouse muscle, was chosen to investigate the use of P(3HHx‐3HO‐3HD‐3HDD) films as a potential scaffold for cardiovascular/skeletomuscular tissue engineering. From the literature, it is understood that C2C12 cells gradually fuse to form myotubes or plurinucleate syncytia. Myotubes then differentiate to attain functional features of a muscle cell (Burattini *et al*., 2004). Bearing in mind the potential application of P(3HHx‐3HO‐3HD‐3HDD) films as cardiovascular/skeletomuscular tissue engineering scaffolds, differentiation of the C2C12 cells into cells of myogenic lineage would be of interest. The MTT assay was used to measure the biochemical activity of the cells seeded on the films. Attachment and proliferation of C2C12 cells on the film samples were studied over a period of 1, 3 and 7 days. Standard tissue culture plastic was used as the positive control for this experiment. The results of these biocompatibility studies have been summarized in Fig. 8.

The growth of C2C12 cells on both film samples and the TCP was comparable at the end of the 1st day of seeding. The % cell proliferation on the film samples was 101 ± 0.3% compared to the TCP at the end of day 1. It is known that the cells attach to the surface of the substrate or scaffold by focal contacts. At the end of day 3, there was a gradual increase in cell growth on the P(3HHx‐3HO‐3HD‐3HDD) film samples. The % cell proliferation on the film samples was 119 ± 0.43. However, there was a significant increase in the growth of the C2C12 cells on the P(3HHx‐3HO‐3HD‐3HDD) film samples compared to the standard TCP at the end of day 7. The % cell proliferation was found to be 172 ± 0.05% (*P = 0.0129). The % cell proliferation on day 7 was also significantly higher in comparison with % cell proliferation on day 1 (*P = 0.0137). The data confirmed that P(3HHx‐3HO‐3HD‐3HDD), an mcl‐PHA copolymer produced using biodiesel waste as the sole carbon source, supported the adhesion and proliferation of C2C12 cells. In a study conducted by Bagdadi *et al*. (2016), % cell proliferation of 21.11 ± 5.29% was observed when C2C12 cells were cultured on neat P(3HO) films for 24 h (Bagdadi *et al*., 2016). In another study, human keratinocyte cell line (HaCaT) was cultured on the neat P(3HO) film samples (Rai *et al*., 2011a,b). Highest % cell proliferation of 73.09% was obtained after 7 days of incubation (Rai *et al*., 2011a,b). Hence, it is known that PHAs are able to support cell adhesion and proliferation and are hence highly biocompatible (Rai *et al*., 2011a,b; Basnett *et al*., 2013). Data obtained in this study were consistent with the reports cited in literature by several other researchers (Misra *et al*., 2006; Basnett *et al*., 2013). Hence, from this particular study, it has been established that the PHA produced using unprocessed biodiesel waste as the carbon source was suitable to be used as a substrate for mammalian cell growth and hence as a scaffold material for soft tissue engineering.

#### Scanning electron microscopy

C2C12 cells were seeded onto the P(3HHx‐3HO‐3HD‐3HDD) film samples. The cells were fixed and viewed under the SEM at day 3 and 7 as shown in Fig. 8. For this particular study, C2C12 cells were cultured in basal media (DMEM media with 10% fetal calf serum), and therefore, these cells were not expected to undergo differentiation. SEM images revealed the adhesion of C2C12 cells on the P(3HHx‐3HO‐3HD‐3HDD) film samples on day 3. The film samples were covered with a confluent layer of cells by day 7, a confirmation of cell proliferation and growth. This also confirmed the results of the MTT assay. The morphology of the cells transitioned from cuboidal shape on day 3 to elongated cells on day 7. From the literature, it is known that the transition in cell shape is indicative of myogenic differentiation and is usually followed by the assembly of structures (actin and myosin filaments) involved in contraction (Burattini *et al*., 2004). This will be investigated in future by culturing C2C12 on P(3HHx‐3HO‐3HD‐3HDD) films in low serum differentiation media. Presence of confluent, elongated C2C12 cells on P(3HHx‐3HO‐3HD‐3HDD) films confirmed that it was biocompatible and supported cell growth and proliferation.

In conclusion, a novel mcl‐PHA, P(3HHx‐3HO‐3HD‐3HDD) was produced using unprocessed biodiesel waste as the sole carbon source. This finding could lead to the use of inexpensive substrates such as biodiesel waste as a replacement for expensive commercially available carbon sources for PHA production, paving the way for its economical production and hence successful commercialization. This study successfully demonstrated that P(3HHx‐3HO‐3HD‐3HDD) was highly biocompatible in nature and supported cell adhesion and proliferation. Due to its amorphous nature, P(3HHx‐3HO‐3HD‐3HDD) could also be used as an additive or a compatibilizer to obtain suitable blends and composites, further extending their application in the field of tissue engineering. Hence in this work, a completely novel mcl‐PHA with great potential in soft tissue engineering was produced using unprocessed biodiesel waste, converting waste to a high‐value medical product.

## Experimental procedures

### Bacterial strains, media and culture conditions

*Pseudomonas mendocina* CH50 was obtained from the National Collection of Industrial and Marine Bacteria (NCIMB). They were cultured using sterile nutrient broth medium at 30°C at 200 rpm for 16 h in a shaking incubator. Stock cultures were stored in 40% glycerol at −80°C.

### Cell lines, media and culture conditions

C2C12 (myoblast) cell line was obtained from the culture collection of the University of Westminster, London, UK. They were cultured using Dulbecco's modified Eagle's medium (DMEM) at 37°C at 5% CO_2_ in a humidified incubator.

### Chemicals

Chemicals used in this study were purchased from Sigma‐Aldrich (Dorset, UK) and VWR (Poole, UK). Chemicals for the cell culture studies were obtained from Lonza (Slough, UK). Live versus Dead staining kit was purchased from Life Technologies, Paisley, UK. Biodiesel waste was obtained from a company called Purefuels, London. They convert waste frying oil into biodiesel via the alkaline esterification method.

### Production of PHAs by *Pseudomonas mendocina* CH50 using 50 g l^−1^ of biodiesel waste as the sole carbon source

Production of PHAs by *P. mendocina* CH50 using 50 g l^−1^ of biodiesel waste (crude glycerol phase) was carried out in 5 l bioreactors (320 series Fermac, Electrolab, Tewkesbury, UK). The working volume used in this study was 3.5 l. Fermentation was carried out in batch mode in two stages. The seed culture was prepared using a single colony of *P. mendocina* CH50 to inoculate sterile nutrient broth. This was incubated for 16 h at 30°C, 200 rpm. Ten per cent (v/v) of the inoculum was used to inoculate the second stage seed culture (mineral salt medium‐MSM) which was incubated at 30°C, 200 rpm for 24 h. This stage is also known as the acclimatization stage. Ten per cent (v/v) of the second stage seed culture was used to inoculate the final PHA production media (MSM media; Basnett *et al*., 2012; Rai *et al*., 2011a,b). All the media components including the biodiesel waste were sterilized at 121°C for 15 min prior to inoculation. The air flow rate was set at 1vvm, and the stirrer speed was set to 200 rpm. pH of all MSM media components, including biodiesel waste, was adjusted to 7.0 at the beginning of the fermentation. The culture was grown for 48 h, at 30°C, 200 rpm. Temporal profile of the production of PHAs by *P. mendocina* CH50 using 50 g l^−1^ of biodiesel waste phase was obtained by monitoring various parameters such as optical density, biomass, nitrogen and carbon concentration at regular intervals throughout the course of the fermentation.

### Optical density measurements, biomass estimation and nitrogen estimation

One millilitre of culture was withdrawn after every 3 h in triplicates during the course of the fermentation. O.D. measurements were carried out at 450 nm using Spectrophotometer SB038 (Cadex, Richmond, Canada) and recorded to monitor the growth of the organism. During profiling, 1 ml of sample was withdrawn in a preweighed Eppendorf tubes and centrifuged at 12,000 rpm for 10 min. Pellet obtained was washed with water, then washed with 10% ethanol and centrifuged at 12,000 rpm (Heraeus Multifuge X3R Centrifuge, Thermo Scientific) for 10 min to remove impurities. The pellet obtained was freeze‐dried. The weight of the Eppendorf tubes containing the dried biomass was measured, and the difference was recorded as the biomass weight. The cell pellet obtained after centrifugation was freeze‐dried and weighed. The supernatant obtained after centrifugation was used to measure the pH and also analyse the amount of nitrogen, in the form of ammonia ions, using the phenol hypochlorite method (Weatherburn, 1967).

### Total carbon estimation using the chemical oxygen demand

Closed reflux titrimetric analysis was carried out to determine the chemical oxygen demand (COD) of the samples as described in Environment Agency (UK) Standard method 5220D. Two millilitres samples were used for the analysis. Diluted samples were added to Ficodox (3.8 ml), which is a mixed COD reagent. This mixture was treated on a preheated heating block for 1.5 h at 150°C in closed digestion tubes. A 0.025 M ferrous ammonium sulfate (FAS) was used as the titrant along with 2‐3 drops of Ferroin indicator solution (Fisher Scientific, Loughborough, UK). This solution was used to titrimetrically determine the residual potassium dichromate present in the Ficodox digestate following the digestion of the sample. The COD deduction was calculated using the following equation:

$$\text{COD}\left(\text{mg}\;l^{-1}\right)=\left(V_{b}-V_{s}\right)*\text{DF}*M*4000$$

where *V*_b_ and *V*_s_ are ferrous ammonium sulphate (FAS) titrant volumes for the blank and the sample respectively, DF is the sample dilution factor and M is the molarity of FAS titrant (Fernando *et al*., 2012).

### Polymer extraction from biomass

The polymer was extracted from the dried biomass by using chloroform/sodium hypochlorite dispersion method (Hahn *et al*., 1994). Lyophilized biomass was placed in conical flasks. Sodium hypochlorite solution and chloroform were added into biomass and incubated for 2 h at 30°C in the orbital shaker (140 rpm). After incubation, the slurry was centrifuged for 20 min at 3900 rpm (Heraeus Multifuge X3R Centrifuge, Thermo Scientific) to allow phase separation. The organic layer containing chloroform and dissolved polymer was collected, filtered and concentrated. The polymer was precipitated using ice‐cold methanol. Polymer mass fraction was calculated as a percentage of the cell dry mass, using the formula:

$$\begin{array}{cc} & \text{Polymer mass fraction}(\%\text{DCM})\\ & =(\text{polymer weight/biomass}*100) \end{array}$$

### Characterization of the PHAs produced

#### Gas chromatography mass spectrometry

Monomeric composition of the PHA produced using both biodiesel waste as the sole carbon source was identified using GC‐MS as described in Furrer *et al.,* 2007. Prior to the GC‐MS analysis, polymer samples were methanolysed. GCMS analysis was carried out using a Varian GS/MS system consisting of Chrompack CP‐3800 gas chromatograph and Saturn 200 MS/MS block.

#### Nuclear magnetic resonance

Structural identification of the polymer was carried out using ^13^C and ^1^H NMR spectroscopy (Bruker Avance III 600 Cryo). Twenty milligrams of the purified polymer sample was dissolved in 1 ml of deuterated chloroform (CDCl_3_) and analysed. All NMR spectra were measured at 298 K. Solution ^1^H and ^13^C NMR spectra were recorded using Bruker NMR spectrometer AVANCE III 600 (Coventry, UK) equipped with a ^1^H,^13^C‐cryoprobe. Data acquisition and processing were performed using standard Bruker topspin (version 2.1) software. ^1^H and ^13^C chemical shifts were calibrated using residual solvent peak (^1^H 7.26 ppm, ^13^C 77.15 ppm for chloroform). This was carried out at University College London, UK (UCL, Department of Chemistry).

#### Differential scanning calorimetry

Thermal properties such as the melting temperature (*T*_m_) and glass transition temperature (*T*_g_) of the PHA produced using biodiesel waste were determined using DSC (DSC 214 Polyma, Netzsch, Germany equipped with Intracooler IC70 cooling system). Five milligram of the polymer sample was used for this analysis. Samples were heated, cooled and then heated again between −50°C and 100° at a heating rate of 20°C min^−1^. Obtained thermograms were analysed using proteus 7.0 software.

#### Preparation of solvent cast PHA films

One gram of the PHA produced using biodiesel waste as the carbon source was dissolved in 10 ml of chloroform, and the PHA solution was mixed thoroughly before being poured into glass petri dishes. They were allowed to dry at the room temperature (Basnett *et al*., 2013)**.** Films were stored for 3 weeks at room temperature prior analysis.

### Characterization of the solvent cast PHA films

#### Scanning electron microscopy

Surface topography of the PHA films was studied using SEM. The film samples were mounted on aluminium stubs and gold‐coated before being viewed under SEM (JEOL 5610LV‐SEM). This study was carried out at the Eastman Dental Hospital, University College London.

#### Surface roughness

Surface roughness of the PHA film samples was measured using a laser‐based optical non‐contact 3D profilometer (Sony Proscan 1000 Laser Profilometer, Tokyo, Japan). This was carried out at the Eastman Dental Institute, University College London, UK.

#### Water contact angle measurements

Static contact angle study was carried out to measure the hydrophobicity of the PHA films. This was carried out using the KSV Cam 200 optical contact meter as described in Basnett *et al*., 2013. Ten microlitres of water was added on the surface of the sample, and a total of 10 images were captured with a frame interval of one second. This experiment was carried out in triplicates. This analysis was carried out at the Eastman Dental Institute, University College London, UK.

#### Protein adsorption assay

Protein adsorption tests were carried out by incubating 1 cm^2^ film samples in 400 μl of undiluted fetal bovine serum (FBS) at 37°C for 24 h in the 24‐well tissue culture plate. After incubation, the samples were rinsed three times with phosphate buffer saline (PBS) and incubated in 1 ml of 2% sodium dodecyl sulphate (SDS) in PBS, for 24 h, at room temperature, under vigorous shaking. This was carried out to extract proteins attached to the surface of the polymer. The overall concentration of the proteins adsorbed on to the PHA film samples was quantified using the Bicinchoninic acid assay (Rai, 2010). This assay was carried out in triplicates.

### *In vitro* biocompatibility tests

#### Cell seeding and proliferation studies

C2C12 cells (cell density – 25,000 cells ml^−1^) were seeded on to the UV sterilized PHA films. They were cultured for a total period of 7 days. Cell viability tests were carried out at the end of day 1, day 3 and day 7 using the MTT assay. Standard tissue culture plastic was used as the positive control.

$$\%\text{Cell Proliferation}=\frac{\text{Mean absorbance of sample}}{\text{Mean absorbance of control}}*100$$

#### Scanning electron microscopy

C2C12 seeded on the PHA films was fixed in 2% paraformaldehyde. These cell‐seeded films were dehydrated; gold‐coated and viewed using SEM.

## Conflict of Interest

None declared.
